# Assessing the per Capita Food Supply Trends of 38 OECD Countries between 2000 and 2019—A Joinpoint Regression Analysis

**DOI:** 10.3390/life13051091

**Published:** 2023-04-27

**Authors:** Tímea Csákvári, Diána Elmer, Noémi Németh, Márk Komáromy, Luca Fanni Kajos, Bettina Kovács, Imre Boncz

**Affiliations:** 1 Institute of Health Insurance, Faculty of Health Sciences, University of Pécs, 7621 Pécs, Hungary; 2 National Laboratory on Human Reproduction, University of Pécs, 7624 Pécs, Hungary

**Keywords:** food supply, calories, fat, protein, trend, OECD

## Abstract

Food supply has an impact on the prevalence of diet-related non-communicable diseases. We aimed to analyze the protein, fat (g/capita/day) and calorie (kcal/capita/day) supply from 2000 to 2019 as derived from the OECD Health Statistics database. A joinpoint regression was used to examine the number and location of breakpoints in the time series. The annual percent change (APC) was calculated using Joinpoint 4.9.0.0. The per capita daily kcal per nutrient was calculated for each country and the resulting percentage distributions were compared to the acceptable macronutrient distribution ranges. Protein, fat and calorie supplies have increased significantly between 2000 and 2019. Each started to show a much steeper, positive change between 2012 and 2014 (APC_fat_: 1.0; 95%CI: 0.8–1.1; APC_protein_: 0.5; 95%CI: 0.3–0.6; APC_kcal_: 0.4; 95%CI: 0.3–0.5). In terms of the composition of the daily calorie intake per capita, the overall share of fat (+4.9%) and protein (+1.0%) increased between 2000 and 2019. We found significant differences among countries and also an increasing and optimal proportion of consumed protein per total calorie in all countries over the last two decades. We concluded that several countries have access to fat availability above the optimal level, which deserves particular attention from health policy makers in the fight against obesity and diet-related diseases.

## 1. Introduction

The prevalence of non-communicable chronic diseases is increasing, and such diseases can cause a significant decline in quality of life, loss of function, years of life lost and premature death, even in the population under 65 years of age. Although the premature mortality rate in the European Union has been on a declining trend in recent decades, there is still significant geographical inequality between the countries that joined before and after 2004 [1]. A further worrying statistic is that the probability of dying from one of the four main chronic diseases between the ages of 30 and 70 is 11.65% (95%CI: 8.95–14.80) on average for the member states of the Organisation for Economic Co-operation and Development (OECD) [2].

One of the common features of the diseases mentioned above is that a significant proportion of them are preventable, while the quality of life and life expectancy could be improved and extended with a healthy lifestyle. Risk factors include alcohol consumption, smoking and physical inactivity. Another contributing factor, and the focus of this study, is eating an inadequate amount and/or quality of food, which some research suggests may be responsible for up to 80% of the development of chronic diseases [3,4].

Diet-related diseases were responsible for around 11 million deaths worldwide, accounting for 22% of all deaths, in 2017, indicating a risk factor even greater than that associated with mortality related to smoking [5]. The closely related problem of obesity is also a steadily growing public health problem. The World Health Organization (WHO) reports that the proportion of obese people has tripled since 1971, reaching 39% of adults and 13% of the total population in 2016 [6].

The increasing prevalence of obesity worldwide reflects an increasingly “obesogenic” dietary environment and long-term changes in activity levels and energy expenditure [7].

The Thirteenth General Programme of Work (GPW13) shows the way forward for the WHO’s work in the period of 2019 to 2023. This includes efforts to reduce salt intake and eliminate trans fatty acids to promote a healthier lifestyle and the overall well-being of all ages [8].

One can prevent developing several chronic diseases by following a diet consisting of a healthy quantity and quality of food. For example, studies suggest that regular consumption of fruit and vegetables is protective against certain types of cancer and cardiovascular diseases [9,10].

Overall, in addition to changes in consumption patterns, there is a global change in the food supply itself. The nutrition transition, which refers to the shift in developing countries to a Western-style, lower-quality diet alongside/after economic development, is one of the proven causes of the increasing prevalence of overweight and obesity, alongside insufficient physical activity, and thus, a consequence of energy imbalance [11,12]. One study estimates the global average energy intake to be 3430 kcal by 2030, rising to an average of 3490 kcal by 2050. The same study describes a significant difference between developed and developing countries, although projected to decrease (+570 kcal in developed countries in 2030, +490 kcal in 2050) [13].This can lead to food insecurity, which refers to the presence of a diet containing an inadequate quality and/or quantity of nutrients and can negatively impact an individual’s health. Moradi et al. point out that food insecurity can include not only quantitative but also qualitative hunger, leading to both malnutrition and obesity in the long term. As a result, it can be a possible risk factor in both developing and developed countries [14].

However, the global shift can also have a direct impact on the development of T2DM, cardiovascular disease and cancer, independently of obesity [15]. Consumption of sugary products and higher intakes of saturated and trans fats [16], on the other hand, have been linked to type 2 diabetes mellitus (T2DM) [17,18]. More importantly, even just exposure to sugar is a risk factor for T2DM [19].

The easier accessibility of unhealthy food is directly linked to the obesogenic diet, especially among the poorer population [20]. Another study by Smith et al. shows that the easier availability of unhealthy food among adolescents has a negative, albeit small, impact on eating habits, with students being more likely to choose unhealthier alternatives for their daytime meals if the grocery/takeaway is located at a shorter distance from their school [21].

A significant relationship between food energy supply and average body weight gain has also been described in high-income countries [22]. Basu et al. find that increasing the per capita daily energy intake by 150 kilocalories of sugar increases the prevalence of diabetes by 1.1% [19].

A recent study shows that changes in the quantity of food available for consumption are also able to result in malnutrition or overnutrition, which may further lead to increased mortality among certain age groups. For instance, a 10% increase in the amount of protein available for consumption reduces mortality, while a decrease in the amount of protein available for consumption increases mortality. A reduction in the proportion of carbohydrates in the diet of people over 25 years of age increases the risk of mortality. Furthermore, an increase in the proportion of fat also increases the risk of mortality in the same age group [23,24].

Adequate energy intake and adequate macronutrient intake in the diet, in line with international recommendations, are essential for the prevention of diet-related diseases, as they contribute to maintaining an optimal body weight. The main macronutrients are proteins, fats and carbohydrates, and in some cases, fiber is either included or excluded from carbohydrates. Different international recommendations are available for the correct proportions of the three main macronutrients within the total energy intake, with slightly different ranges [24,25]. Long-term dietary intake of both below and above the optimal ranges can lead to poor health. Consumption below the lower end of the ranges can increase the risk of certain deficiency diseases and conditions (kwashiorkor, marasmus, hypoglycemia, etc.), while excessive intake may increase the risk of developing certain diet-related diseases such as hypertension, obesity, type 2 diabetes or even cancer.

Considering the results of the above-mentioned studies, we assumed that the amount of food and nutrient supply available in each country influences the prevalence of diseases linked to overnutrition. Therefore, to support our understanding of the risk factors influencing diet-related diseases, our analysis aimed to analyze the available fat, protein and calorie per capita supply among the OECD countries.

It is important to emphasize that these indicators do not represent the actual quantity consumed, but rather the amount available for consumption in each country, with the latter being an overestimation of food intake [26]. Food supply has already been examined in other studies in the context of improving health, both at the national and international levels [27,28,29,30,31]. The main advantage of the Food Balance Sheets (FBS) over other data collections that measure consumption directly or indirectly is that they are available to everyone, have long time series for many countries [13] and are therefore used as a proxy for food consumption. The data do not include the quantity not intended for human consumption (feeding of farm animals, production of other non-food products, etc.), however, and the FBS do not consider food waste at the household- and retail-level or during distribution. As a result, there are some limitations when using the FBS for assessing food consumption patterns, as food supply is not equal to intake [32].

We aimed to complement these studies with comparisons between OECD member countries.

## 2. Materials and Methods

### 2.1. Sources of Data

In our analysis, we assessed the food supply-related indicators for 38 OECD member countries from 2000 to 2019.

The main objectives of the OECD, highlighting the ones in the focus of our study, include promoting policies that “promote sustainable economic growth” and the efficient “use and development of their resources” [33]. Of the 38 member states analyzed, not all were members during our selected period. To gain a broader understanding of the global situation and to support the above-mentioned objective of common development among the countries, we included all the countries that were member states in 2022 and for which data were available in the OECD database for the period in question.

For this purpose, the daily per capita protein (g/capita/day), fat (g/capita/day) and calorie (kcal/capita/day) intake were analyzed at the national and international levels. The data were derived from the OECD Health Statistics database, with the original source being calculations made by the Food and Agriculture Organization of the United Nations (FAO)’s Food Balance Sheets. For the indicators shown, the amount reported as the “Grand Total” was analyzed. Quantitative data on the food supply available for consumption for all raw/unprocessed and processed products were calculated by examining the corresponding food components, which were then divided by the number of people involved in food consumption (i.e., residing in the country) during the period. A joinpoint regression analysis was applied to these indicators, the procedure for which is described in the following subsection.

Based on the available data, the per capita daily kcal requirement per nutrient was also calculated for each country using the Atwater coefficient (4 kcal/g for protein, 9 kcal/g for fat) [34]. The amount of carbohydrates (and fiber) was determined by subtracting the energy value of these two nutrients from the total kcal and then determining the percentage distribution of these three nutrients. The results obtained were checked against published data from other studies, which were found to be in line with our results [35]. The resulting percentage distributions were also compared with the acceptable macronutrient distribution ranges [25].

For each indicator examined, we also calculated the average trend for the OECD member countries, with the latter being defined as the average of the countries providing the data (*n* = 38).

### 2.2. Statistical Methods

To characterize the trends of the above-mentioned indicators and their changes among the selected countries, a joinpoint regression analysis was used, with the help of which we aimed to identify the number and location (years) of breakpoints within the time series. The essence of a joinpoint regression analysis is that one does not just calculate a linear line for the time series, but rather identifies the major joinpoints in the time series where the slope or even the direction of the trend line changes. The software used in the analysis automatically performs a trend analysis on the input data for 0–3 breakpoints (i.e., runs three different models) and then selects the most optimal model. This explains the different number of trends obtained in the analysis for each unit (country) or time series. The choice of the maximum number of possible breakpoints was based on the Joinpoint regression program’s recommendations, as was the choice of the model on which the analysis was based (data-driven, weighted Bayesian information criterion (WBIC)) [36]. As stated in a recommendation of the Effective Practice and Organisation of Care (EPOC) group, a similar statistical method, an interrupted time series analysis, can be conducted when there are at least three time points before and three after an intervention (in this case: joinpoint) to define two or more regression lines—therefore, we determined a minimum number of three years within a trend [37,38].

As a result, two main indicators were calculated during our analysis. The first was the annual percentage change (APC), which shows the relative change in one indicator in a country within the shorter trends determined by the joinpoint regression model. The second was the annual average percentage change (AAPC), characterizing the whole time series (2000–2019). The most important benefit of these indicators is the ability to render the variables measured in different dimensions comparable.

The difference in the slope of each trend was tested using a t test, with significant differences marked in all cases (*p* < 0.05) and confidence intervals at a 95% probability level. The analysis was performed using Joinpoint 4.9.0.0 software [39]. Our detailed results showing the breakpoints and trends of each country for the three selected indicators can be seen in the Supplementary Materials.

## 3. Results

The main results of the joinpoint regression analysis of the OECD averages are summarized in Table 1. The average per capita protein, fat and calorie supplies increased significantly between 2000 and 2019. The average available quantity of fat increased at the fastest rate (0.6%/year), followed by protein (0.3%/year) and kcal (0.2%/year). The more significant rises seen in the early 2000s are shown, creating breakpoints in the time series around 2006 and 2007, where the per capita levels of nutrients and calories started to stagnate (fat, calories) or slightly decrease (protein). Afterwards, an even steeper and significant upward trend for all three indicators is seen, starting approximately around 2012–2014.

### 3.1. Fat Supply

**Figure 1:**
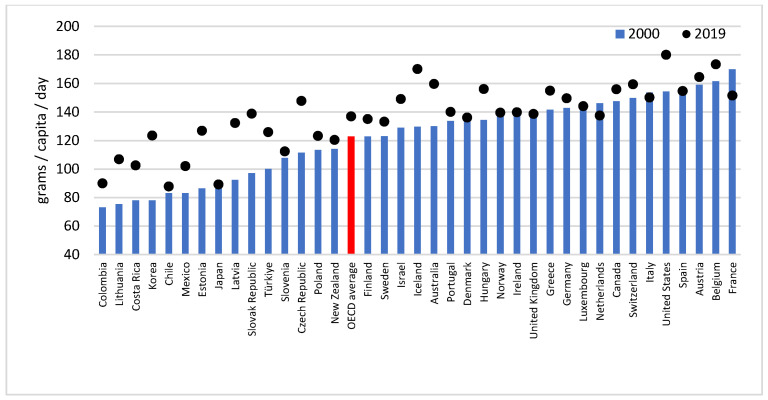
Fat supply of the 38 OECD countries in 2000 and 2019.

In 2000, the average daily fat intake per capita in the OECD member states was 122.8 g, increasing by 5.13% in 2010 (129.1 g) and by a further 6.0% (136.9 g) in 2019. In 2000, France had the highest value (169.9 g) and Colombia the lowest (73.2 g). There was a more than twofold difference between the lowest (Chile: 87.8 g/capita/day) and the highest (United States: 180.1 g/capita/day) values in the last year assessed (Figure 1). Between 2000 and 2010, the largest increases in the fat supply were observed in Latvia (+29.87%), Lithuania (+29.84%) and Korea (+29.32%), while between 2010 and 2019, in Estonia (+37.93%), Korea (+22.28%) and Slovakia (+19.86). By contrast, the Netherlands (−14.50%), Spain (−7.41%) and Ireland (−6.58%) decreased the most in the first, and Slovenia (−7.72%), France (−6.88%) and Austria (−5.57%) in the second ten years assessed.

In 52.6% of the countries studied (*n* = 20), we observed a significant positive trend in the per capita fat supply, with the highest rate seen in Korea (AAPC: 2.6; 95%CI: 2.4–2.7). Only in France did we see a significant downward trend (AAPC: −0.8; 95%CI: −0.9–−0.6). Overall, the OECD average also showed a steady increase (AAPC: 0.6; 95%CI: 0.5–0.7). We found two breakpoints in the time series studied, creating three major trends. The first (APC: 0.7; 95%CI: 0.5–0.9) ended in 2006. Afterwards, until 2013, no significant trend could have been identified. Then, from 2013 onwards, there was a sharp increase in the amount of the fat supply (1.0; 95%CI: 0.8–1.1) (Table S1).

### 3.2. Protein Supply

The international average of the available protein supply has increased significantly over the past two decades (AAPC: 0.3; 95%CI: 0.2–0.4), reaching 103.8 g/capita/day in 2019, 4.6 g more than in 2000. Two breakpoints were found in the time series as well, revealing three trends. Until 2008, the average change in the protein quantity per capita was 0.4% (95%CI: 0.2–0.5), although this rate became more moderate until 2012. The highest positive rate for the whole time series was observed after 2012 (APC: 0.5; 95%CI: 0.3–0.6) (Table S2).

**Figure 2:**
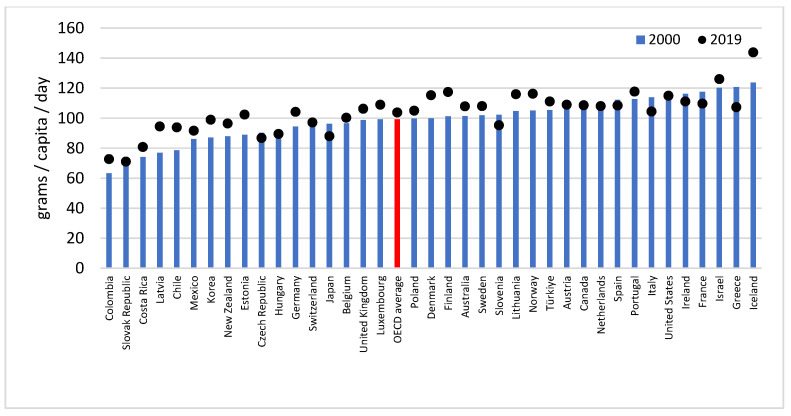
Protein supply of the 38 OECD countries in 2000 and 2019.

The supply rate also increased in 14 countries and decreased in 7 (France, Greece, Italy, Japan, Slovakia, Slovenia and Spain). The highest amount in the first year belonged to Iceland (123.7 g), the lowest to Colombia (63.4 g). In 2019, the lowest rate was observed in Slovakia (71.1 g/capita/day), while the highest rate, more than double, was also observed in Iceland (143.9 g/capita/day). The largest relative change between 2000 and 2010 was found in Latvia (+25.06%), Norway (+11.89%), Luxembourg (+11.19%), Hungary (−10.64%), Japan (−9.54%) and Greece (−7.45%). Somewhat smaller changes were seen in the amount of the available protein supply in 2010 and 2019. Growth was shown as high as 18.79% in Colombia, 11.98% in Iceland and 10.47% in Chile, while the lowest were present in the Czech Republic (−6.47%), Italy (−6.45%) and in Slovenia (−5.17%) (Figure 2).

### 3.3. Calorie Supply

**Figure 3:**
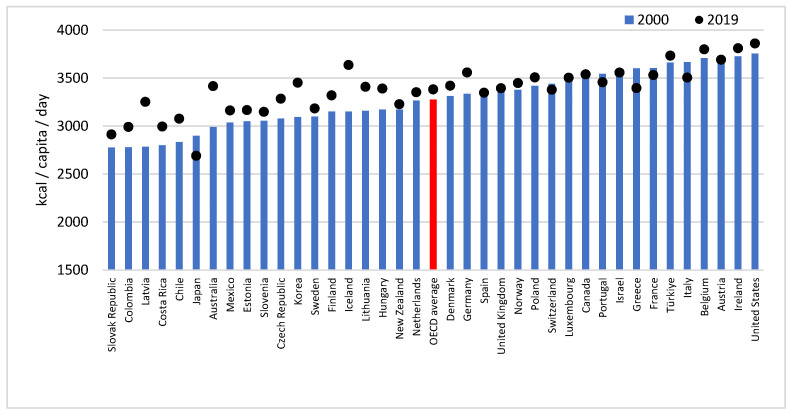
Calorie supply of the 38 OECD countries in 2000 and 2019.

The international average daily calorie intake per capita also showed a significant increasing trend between 2000 and 2019 (AAPC: 0.2; 95%CI: 0.1–0.2). Similar to what was seen in the fat supply, it increased continuously (APC: 0.2; 95%CI: 0.1–0.3) until 2006 and then again, even more sharply, from 2014 onwards (APC: 0.4; 95%CI: 0.3–0.5) (Table S3). A total of 13 members have also shown significant increases over the last 20 years, while 2 countries (Japan and Italy) have shown decreases. Until 2010, Australia (+14.01%), Latvia (+12.60%) and Estonia (+8.36%) had the largest average kcal increase per capita, and Japan (−7.59%), Spain (−5.45) and Greece (−4.44%) the most significant decrease. In the second period (2010–2019), the endpoints of our sample consisted of Hungary (+10.74%), Colombia (+10.49%), Iceland (+7.54%), Estonia (−4.18%), Italy (−2.18%) and Switzerland (−2.00%). In 2019, the two endpoints were Japan (2691 kcal/capita/day) and the United States (3862 kcal/capita/day), while the OECD average was 3382 kcal/capita/day in the same year (Figure 3).

The distribution of the daily calorie intake has also changed between 2000 and 2019, as shown in Table 2. In 2019, the level of fat intake ranged from 27.7% (Japan) to 48.6% (Iceland), the distribution of protein from 10.2% (Slovakia) to 18.3% (Iceland), and carbohydrate from 33.2% (Iceland) to 60.4% (Colombia). The largest increase in protein per year between the first and the last year studied was in Latvia (+17.2%), while the largest decrease was found in France, with −5.5%. If we compare the results with the recommended distribution, we find that in 2000, 17 states (44.7%) still had a lower (*n* = 1) or higher (*n* = 16) distribution for fat than the recommended one (Table 2).

Protein was lower in only four countries, carbohydrate was higher in six countries and only one country (France) had a lower (only marginally) proportion. In comparison, the amount of fat consumed, and thus its share of daily calories, seems to have increased by 2019, with 27 member countries now having a share above the upper limit of 35% of the recommended intake. At the same time, however, all the countries surveyed were within the limits for protein, with no deviation. The carbohydrate intake also showed a downward trend, with the average intake of six countries falling below the lower limit of 45% in 2019, while the average of none of the countries exceeded the upper limit.

## 4. Discussion

In our study, we estimated the changes in the amount of fat, protein and calories available in each OECD country and the ratio of each macronutrient (fat, protein, carbohydrate) between 2000 and 2019.

Our results show that the available calorie supply increased significantly. Significant differences were also found between the trends in individual member states. Possible reasons for the increase in food quantity include the globalization of food markets and policies that support agricultural technology developments and productivity and infrastructure [40]. Out of the assessed macronutrients, the steepest trend was observed for fat (APC: 0.6; 95%CI: 0.5–0.7). This can be explained by the spread of the Western diet, which includes a greater proportion of processed and higher-fat foods [31,41]. The fat content within the proportion of macronutrients also grew from 32.7% (95%CI: 30.7–34.7%) to 37.6% (95%CI: 36.0–39.3%). Together, we saw an increase in 30 countries, with the highest rate seen in Korea (AAPC: 2.6; 95%CI: 2.4–2. 7), and only 8 member states have shown stagnation or decline between 2000 and 2019 (Japan, Ireland, UK, Luxembourg, Netherlands, Italy, Spain, France). Based on the results shown, we draw the attention of health policy makers to the health risks associated with the spread of high-fat diets, such as the increased incidence of obesity and cardiovascular diseases. If the GPW13 and other action plans targeting these diseases are to be effective, these trends must also be given particular attention.

The increasing food supply is a consequence of the ever-growing global population and demand. However, in addition to food security, this trend can also negatively affect developed and developing countries by affecting the quantity and quality of nutrients available and, consequently, consumed. In this context, the nutrition transition can have a negative impact on obesity, but also on the morbidity of cardiovascular diseases and certain types of cancer [40].

Our results confirm the need and justification for efforts to focus on (further) improving the lifestyle of the population. Schmidhuber et al. also conclude that there is a global increase in fat and protein, causing a corresponding decrease in carbohydrates [35]. Although our own results were slightly lower due to differences in the population and methodology, they were nevertheless consistent with their results.

Austin et al. describe an overall increase in the daily calorie intake between 1970 and 2006 in the population they studied, with the largest increase seen in obese people, confirming the link between calorie intake and obesity. In addition, different macronutrient distributions are observed for individuals in different weight categories, indicating a strong inverse relationship between an increase in the proportion of protein in the daily calories and the daily energy intake in the sample. Regardless of body weight, they find that a 10% increase in the ratio of protein in an obese individual can result in a 438 calorie reduction in the energy intake when replacing it with carbohydrates and 620 calories when replacing it with fat. This suggests that, while maintaining an optimal level of daily calorie intake in the population, it is important to, if necessary, increase the recommended proportion of protein at the expense of either carbohydrate or fat [42,43]. Reducing carbohydrates, for example, contributes to an increase in energy expenditure and thus to maintaining an ideal body weight [44]. However, diets that are higher in protein but lower in carbohydrates may even increase the risk of colorectal disease [45]. It is important to highlight the benefits of high-carbohydrate, low-fiber and low-fat diets in the context of overall calorie restriction leading to weight loss, which can prevent diabetes among high-risk individuals [46].

The power of our study lies in the fact that we studied food supply trends over a long period and covered 38 OECD member states. We aimed to contribute to the fight against the high incidence and prevalence of diet-related diseases by showing the trends of the available macronutrient supplies.

To the best of our knowledge, no studies have been conducted about food supply trends in all the current OECD countries regarding the selected period. Therefore, our aim was to gain a broader understanding of the global situation and to support the OECD’s objective of common development among the countries.

Our research outcomes can contribute to the development of the sector and be used by other researchers, especially in the development of national or international policies, action plans and strategies for improving health. It is important for the health care system to have sufficient resources and capacity to prevent and treat public health diseases. Thus, knowing and accurately estimating the expected incidence, prevalence and burden of such diseases is key to adequate and effective care. Our research contributes to these planning processes by describing an important and proven risk factor for diet-related diseases: the macronutrients available for consumption. The trends shown will also allow us to infer changes in conditions resulting from inadequate dietary quality and quantity in each country, and to identify which countries and to what extent dietary intakes differ from international recommendations [25].

Our results can increase awareness among food producers [47] and agricultural and health policy makers [48] of the need to take action. This includes improving attitudes toward healthy food consumption, raising health awareness among the population [49,50,51], and securing resources for the prevention and treatment of nutrition-related diseases. Additionally, we propose a strategy to achieve these objectives by taxing unhealthy foods lacking balanced nutrients and further promoting the consumption of healthy foods and ensuring their accessibility and affordability among the whole population.

In addition to the FBS, there are several options for measuring and estimating consumption data [52]. Each of these databases and measurement methods has advantages and some limitations as well [53]. We chose the FBS because they provide widely available data, have long time series for many countries, and are a helpful tool in estimating the impact of available food supplies on obesity and diet-related diseases.

As a limitation of our research, it is necessary to mention that the data from the Food Balance Sheets may slightly overestimate the amount consumed. However, it makes it possible to illustrate the long-term trends of these indicators and to draw conclusions for the countries studied [35]. According to Swarnamali et al., these data can be used to characterize consumption patterns, because there is evidence of a very strong, positive, significant relationship between supply and BMI, as well as between the prevalence of overweight and obesity and the amount of fat consumed per capita [54]. We did not have specific data on the amount of carbohydrates available, in addition to fat, protein and calories, so we could only estimate. Furthermore, given the data available for us to analyze, we were unable to differentiate the carbohydrate and fiber content when calculating the macronutrient ratios. Furthermore, our data were not suitable for differentiating between the quality of individual macronutrients and ingredients (e.g., saturated fat, unsaturated fat, foods with added or natural forms of sugar, etc.). Therefore, any conclusions on health status drawn from these should be treated with caution. We also did not consider other factors that may influence the prevalence of diet-related non-communicable diseases, such as physical activity levels or the chemical occurrence level of contaminants in food.

It is important to stress that the indicators we studied could have been influenced by several possible factors. Such potential confounders include lack of substitutability, religious and cultural customs and traditions, quality and contamination of the available food, the food policy measures of a given country and the health status of the population (e.g., people with special dietary needs). We were also unable to address regional differences within the country, although the type of residence (rural/urban) and socio-economic status may also have a significant impact on the availability of resources within a given area/social strata. It is also worth noting differences in lifestyles and habits between nations. Influencing them is key to achieving a better public health status, which we have seen in recent years in almost all countries with the introduction of health policies, action plans and regulations [55,56].

Additionally, we emphasize that we did not use individual-based food consumption data, but rather the annual sum of available nutrients and calories in a given country divided by its average population in the same year. The actual values may be affected by socio-demographic and regional differences within the population. It is worth mentioning that there is an initiation for creating a comprehensive database for analyzing food consumption patterns through national surveys of the countries that provide data (FAO/WHO GIFT (Global Individual Food consumption data Tool)), but as of now, it is in need of gathering more data before it can be used for such long-term, retrospective analyses.

The reduction of preventable diseases and their risk factors is also of major importance from a health policy perspective, as there is a significant cost overrun in health care linked to these disease groups, and as the quality of life deteriorates, the working capacity of individuals is reduced, with an overall negative impact on the country’s economy. In the future, policy measures to promote healthy lifestyles and healthy eating habits and overall health literacy should be a priority [57,58].

This could include measures to help people make health-conscious choices (regulating the information content of advertising and packaging), measures to change the market environment (public health product taxes and other special taxes associated with unhealthy lifestyles, or value-added tax (VAT) reductions on healthy foods (e.g., fish)) [7,59], or measures that are not directly linked to the individual but are positive nonetheless (regulating agricultural production) [60].

A possible and nowadays widely used policy measure that can achieve a reduction in non-communicable diseases is the taxation of goods linked to unhealthy lifestyles, a policy that is being applied in a growing number of countries [61,62,63]. These taxes are usually based on the risk factors for chronic diseases (e.g., high-sugar foods and beverages, junk food, alcohol, tobacco, etc.) that are (also) caused by poor lifestyle choices. The literature shows that in international practice, the most popular tax is on sugar-containing foods and beverages, although some countries also impose similar taxes on other ingredients or food groups. According to the European Public Health Alliance, 10 European countries have already reduced VAT on fruit and vegetables to the minimum level set by the European Union [64].

However, it is crucial that these measures first and foremost reinforce and support individuals’ health awareness and responsibility for change [1,65,66].

To support the implementation of food system policies that support sustainable and healthy lifestyles, it is essential to understand how these patterns have evolved and may change in the future [67,68].

Overall, we concluded that raising awareness of the public health problem faced by the population of each member state is crucial, as it may cause overconsumption and inadequate nutrient intake. Given that the available quantities of calories and fat have also increased dramatically in recent years, it is worth paying particular attention to and raising awareness of the potential risks, the importance of eating healthy foods in sufficient quantity and quality and their role in disease prevention.

The WHO’s action plan for non-communicable chronic diseases (NCDs) sets a target for diabetes to halt the rise in prevalence by 2025. It is encouraging that, as a result of international efforts, a reduction in the incidence of T2DM has already been documented in some high-income countries by 2019 [69]. If we want to see a similar trend in mortality rates, more attention must be paid to the prevention of chronic and acute complications and to appropriate patient education, of which the development of conscious eating habits must be an essential part.

## 5. Conclusions

Our analysis assessed the availability of certain nutrients and the rate of change in nutrient availability between 2000 and 2019 among 38 OECD countries. We found that there were differences between countries in the indicators of the nutritional environment. We also observed that, on average across the OECD countries, the largest increase was in the available fat (0.6% per year on average), although the protein (0.3%) and total calories (0.2%) supplies also increased. Each of these indicators typically started to show a much steeper positive change after 2012–2014 (1.0% for fat, 0.5% for protein, 0.4% for calories). In terms of the composition of the daily calorie intake per capita, we have also shown that the overall shares of fat and protein have increased. The increasing proportion of protein in the total calories consumed over the last two decades and the existence of an optimal proportion in all the countries is a development that is surely welcomed. However, the increase in the proportion of fat means that 71% of the countries had access to fat above the optimal ratio in 2019. This deserves particular attention from health policy makers in the fight against obesity and diet-related diseases.

## Figures and Tables

**Table 1:** The annual average percent changes (AAPC) and average percent changes (APC) of protein, fat and calories supplies between 2000 and 2019: Results of joinpoint regression models based on OECD averages.

	AAPC (95%CI)	Trends					
		Trend 1		Trend 2		Trend 3	
		APC (95%CI)	Period	APC (95%CI)	Period	APC (95%CI)	Period
Protein	0.3 * (0.2–0.4)	0.4 ** (0.2–0.5)	2000–2007	−0.2 (−0.5–0.1)	2007–2012	0.5 ** (0.3–0.6)	2012–2019
Fat	0.6 * (0.5–0.7)	0.7 ** (0.5–0.9)	2000–2006	0.1 (0.0–0.3)	2006–2013	1.0 ** (0.8–1.1)	2013–2019
Calories	0.2 * (0.1–0.2)	0.2 ** (0.1–0.3)	2000–2006	0.0 (−0.1–0.1)	2006–2014	0.4 ** (0.3–0.5)	2014–2019

*: *p* < 0.05; **: *p* < 0.001.

**Table 2:** Proportion of macronutrients in the daily calorie supply in the OECD countries (average).

Nutrient		2000	2019	Change	*p* Value
Fat	% of daily kcal supply	32.7%	37.6%	4.9%	<0.001
	(95%CI)	(30.7–34.7%)	(36.0–39.3%)		
Protein	% of daily kcal supply	11.7%	12.7%	1.0%	<0.001
	(95%CI)	(11.3–12.2%)	(12.2–13.1%)		
Carbohydrates (and fiber)	% of daily kcal supply	55.6%	49.7%	−5.9%	<0.001
	(95%CI)	(53.4–57.8%)	(47.9–51.5%)		

## Data Availability

Publicly available datasets were analyzed in this study. These data can be found here: https://stats.oecd.org/Index.aspx?ThemeTreeId=9 (accessed on 27 March 2023).

## Supplementary Materials

The following supporting information can be downloaded at https://www.mdpi.com/article/10.3390/life13051091/s1, Table S1: Results of the joinpoint regression models regarding the fat supply of the 38 OECD member states between 2000 and 2019; Table S2: Results of the joinpoint regression models regarding the protein supply of the 38 OECD member states between 2000 and 2019; Table S3: Results of the joinpoint regression models regarding the calorie supply of the 38 OECD member states between 2000 and 2019.
