# Supplementary material for: Assessing the per Capita Food Supply Trends of 38 OECD Countries between 2000 and 2019—A Joinpoint Regression Analysis
Source: Life (Basel). 2023 Apr 27;13(5):1091. doi: 10.3390/life13051091 (PMC10222698; doi:10.3390/life13051091)
Supplement: Supplementary file 1 [file life-13-01091-s001.zip › Table S1.pdf]

Country	AAPC (95%CI)	Trends							
		Trend 1		Trend 2		Trend 3		Trend 4	
		APC (95%CI)	Period	APC (95%CI)	Period	APC (95%CI)	Period	APC (95%CI)	Period
Australia	1.1* (0.7 - 1.5)	1.5*** (1.2 - 1.7)	2000-2015	-0.1 (-1.9 - 1.8)	2015-2019				
Austria	0.2 (-0.6 - 1.0)	-2.1 (-4.9 - 0.8)	2000-2003	1.8** (1.0 - 2.6)	2003-2011	-1.7 (-4.6 - 1.2)	2011-2015	0.4 (-1.4 - 2.3)	2015-2019
Belgium	0.2 (-0.1 - 0.5)	0.0 (-0.3 - 0.2)	2000-2014	0.9 (-0.2 - 2.0)	2014-2019				
Canada	0.4 (-0.3 - 1.1)	-0.1 (-0.4 - 0.2)	2000-2012	3.2 (-1.4 - 8.0)	2012-2015	-0.2 (-1.7 - 1.2)	2015-2019		
Chile	0.4** (0.2 - 0.7)	0.4** (0.2 - 0.7)	2000-2019						
Colombia	1.3* (1.0 - 1.5)	0.5* (0.1 - 1.0)	2000-2009	1.9*** (1.6 - 2.3)	2009-2019				
Costa Rica	1.6* (0.6 - 2.6)	1.9*** (1.2 - 2.6)	2000-2007	0.6 (-0.3 - 1.5)	2007-2014	5.0 (-0.5 - 10.8)	2014-2017	-0.9 (-6.1 - 4.6)	2017-2019
Czech Republic	1.7* (0.9 - 2.4)	3.0*** (2.1 - 3.9)	2000-2008	-1.0 (-2.4 - 0.4)	2008-2015	3.7* (1.0 - 6.4)	2015-2019		
Denmark	0.1 (-0.2 - 0.3)	-0.5* (-0.9 - -0.1)	2000-2010	0.6** (0.2 - 1.1)	2010-2019				
Estonia	2.1* (0.7 - 3.6)	7.8 (-0.3 - 16.6)	2000-2002	-1.0* (-1.7 - -0.3)	2002-2012	9.2* (0.9 - 18.1)	2012-2015	2.1 (-0.4 - 4.7)	2015-2019
Finland	0.5* (0.2 - 0.9)	0.7** (0.4 - 1.0)	2000-2009	2.1* (0.4 - 3.8)	2009-2013	-0.7* (-1.2 - -0.1)	2013-2019		
France	-0.8*** (-0.9 - -0.6)	-0.8*** (-0.9 - -0.6)	2000-2019						
Germany	0.3*** (0.2 - 0.5)	0.3*** (0.2 - 0.5)	2000-2019						
Greece	0.5* (0.1 - 0.8)	0.8*** (0.5 - 1.2)	2000-2009	-0.6 (-1.3 - 0.1)	2009-2016	1.7 (-0.3 - 3.8)	2016-2019		
Hungary	0.9* (0.5 - 1.4)	0.3 (-0.1 - 1.6)	2000-2013	2.4** (1.2 - 3.7)	2013-2019				
Iceland	1.5* (0.7 - 2.4)	2.4*** (1.7 - 3.1)	2000-2007	-1.7 (-6.7 - 3.6)	2007-2010	2.0*** (1.5 - 2.5)	2010-2019		
Ireland	0.4 (-0.1 - 0.9)	-0.4* (-0.9 - 0.0)	2000-2013	2.4** (0.9 - 3.8)	2013-2019				
Israel	0.6 (-0.1 - 1.4)	2.7** (1.5 - 3.9)	2000-2005	-1.0 (-2.7 - 0.7)	2005-2010	1.2* (0.3 - 2.1)	2010-2017	-2.4 (-7.5 - 2.9)	2017-2019
Italy	-0.1 (-1.0 - 0.7)	0.2 (-0.1 - 0.6)	2000-2012	-2.4 (-7.7 - 3.2)	2012-2015	0.5 (-1.3 - 2.3)	2015-2019		
Japan	0.0 (-0.5 - 0.4)	0.0 (-0.4 - 0.4)	2000-2007	-1.5 (-4.3 - 1.4)	2007-2010	0.5** (0.2 - 0.8)	2010-2019		
Korea	2.6*** (2.4 - 2.7)	2.6*** (2.4 - 2.7)	2000-2019						
Latvia	1.9* (1.2 - 2.5)	8.6** (4.0 - 13.5)	2000-2002	3.5** (1.3 - 5.8)	2002-2006	-1.0* (-2.0 - 0.0)	2006-2012	1.6*** (1.0 - 2.1)	2012-2019

Lithuania	1.8* (1.1 - 2.5)	9.4** (4.2 - 15.0)	2000-2002	2.7** (1.2 - 4.4)	2002-2007	-2.0** (-3.1 - -0.9)	2007-2013	2.5*** (1.7 - 3.4)	2013-2019
Luxembourg	-0.1 (-0.5 - 0.2)	1.2 (-0.2 - 2.5)	2000-2004	-1.5*** (-2.2 - -0.8)	2004-2011	0.4 (0.0 - 0.9)	2011-2019		
Mexico	1.1*** (1.0 - 1.2)	1.1*** (1.0 - 1.2)	2000-2019						
Netherlands	-0.3 (-0.7 - 0.2)	-1.4*** (-1.9 - -0.9)	2000-2012	1.6** (0.5 - 2.7)	2012-2019				
New Zealand	0.1 (-0.2 - 0.4)	0.1 (-0.2 - 0.4)	2000-2019						
Norway	0.0 (-0.5 - 0.4)	2.1*** (1.4 - 2.7)	2000-2005	-1.7 (-4.5 - 1.3)	2005-2008	-0.5*** (-0.7 - -0.3)	2008-2019		
Poland	0.5 (-0.4 - 1.4)	-0.3 (-0.7 - 0.2)	2000-2008	3.4 (-0.9 - 7.9)	2008-2011	-1.1 (-5.3 - 3.1)	2011-2014	1.1* (0.1 - 2.0)	2014-2019
Portugal	0.2 (-0.4 - 0.8)	-0.2 (-1.6 - 1.2)	2000-2004	1.8 (-0.4 - 4.1)	2004-2008	-1.1* (-2.1 - -0.1)	2008-2014	0.8 (-0.2 - 1.8)	2014-2019
Slovak Republic	1.6* (1.2 - 2.1)	0.1 (-0.8 - 1.0)	2000-2009	2.8*** (2.2-3.4)	2009-2019				
Slovenia	0.2 (-0.5 - 0.8)	4.2 (-1.2 - 9.8)	2000-2002	0.4 (-0.5 - 1.3)	2002-2009	-0.8** (-1.2 - -0.4)	2009-2019		
Spain	0.0 (-0.2 - 0.2)	-1.6*** (-2.2 - -1.9)	2000-2004	-0.2* (-0.4 - 0.0)	2004-2014	1.8*** (1.3 - 2.3)	2014-2019		
Sweden	0.3* (0.0 - 0.7)	-0.1 (-0.6 - 0.4)	2000-2007	1.3*** (0.8 - 1.8)	2007-2015	-0.9 (-2.0 - 0.3)	2015-2019		
Switzerland	0.2** (0.1 - 0.3)	0.2** (0.1 - 0.3)	2000-2019						
Türkiye	1.4*** (1.2 - 1.6)	1.4*** (1.2 - 1.6)	2000-2019						
United Kingdom	-0.2 (-0.6 - 0.3)	-2.0* (-3.4 - 0.4)	2000-2003	2.1 (-1.0 - 5.3)	2003-2006	-0.3** (-0.4 - -0.1)	2006-2019		
United States	0.9* (0.4 - 1.3)	1.3** (0.6 - 2.0)	2000-2005	-0.7 (-2.3 - 0.8)	2005-2009	0.5 (0.0 - 1.0)	2009-2016	3.1** (1.5 - 4.8)	2016-2019

*: p<.05; **: p<0.01; ***: p<.001

Supplementary Table S1. Results of joinpoint regression models regarding fat supply of 38 OECD member states between 2000-2019.
