# Supplementary material for: Assessing the per Capita Food Supply Trends of 38 OECD Countries between 2000 and 2019—A Joinpoint Regression Analysis
Source: Life (Basel). 2023 Apr 27;13(5):1091. doi: 10.3390/life13051091 (PMC10222698; doi:10.3390/life13051091)
Supplement: Supplementary file 1 [file life-13-01091-s001.zip › Table S2.pdf]

Country	APC (95%CI)	Trends							
		Trend 1		Trend 2		Trend 3		Trend 4	
		AAPC (95%CI)	Period	AAPC (95%CI)	Period	AAPC (95%CI)	Period	AAPC (95%CI)	Period
Australia	0.3* (0.1 - 0.5)	0.3* (0.1 - 0.5)	2000-2019						
Austria	0.1 (-0.2 - 0.4)	-1.5* (-2.9 - -0.1)	2000-2004	0.5*** (0.3 - 0.7)	2004-2019				
Belgium	0.0 (-0.1 – 0.1)	0.0 (-0.1 - 0.1)	2000-2019						
Canada	0.2 (-0.2 - 0.6)	-0.2** (-0.4 - -0.1)	2000-2012	-1.3 (-3.9 - 1.4)	2012-2015	2.7*** (1.9 - 3.6)	2015-2019		
Chile	0.9* (0.3 - 1.5)	1.9*** (1.2 - 2.5)	2000-2006	-1.8 (-5.5 - 1.9)	2006-2009	1.2*** (0.9 - 1.4)	2009-2019		
Colombia	0.8* (0.5 - 1.0)	-0.2 (-0.6 - 0.2)	2000-2010	1.8*** (1.4 - 2.3)	2010-2019				
Costa Rica	0.6 (-0.2 - 1.3)	-3.2 (-7.3 - 1.1)	2000-2002	1.4** (0.7 - 2.1)	2002-2009	-1.0 (-5.2 - 3.3)	2009-2012	1.5*** (1.0 - 2.1)	2012-2019
Czech Republic	-0.5 (-0.2 - 0.1)	1.6** (0.8 - 2.4)	2000-2005	-1.4*** (-1.8 - -0.9)	2005-2013	-0.1 (-0.5 - 0.1)	2013-2019		
Denmark	0.5** (0.2 - 0.8)	0.5** (0.2 - 0.8)	2000-2019						
Estonia	0.9*** (0.7 - 1.2)	0.9*** (0.7 - 1.2)	2000-2020						
Finland	0.8* (0.7 - 1.0)	1.1*** (1.0 - 1.2)	2000-2014	0.0 (-0.5 - 0.5)	2014-2019				
France	-0.5* (-0.7 - -0.3)	-1.1** (-1.7 - -0.5)	2000-2006	-0.2* (-0.4 - 0)	2006-2019				
Germany	0.5*** (0.4 - 0.6)	0.5*** (0.4 - 0.6)	2000-2019						
Greece	-0.5* (-0.9 - -0.1)	-0.7*** (-0.9 - -0.5)	2000-2016	0.8 (-1.6 - 3.2)	2016-2019				
Hungary	0.1 (-0.4 - 0.6)	0 (-0.8 - 0.9)	2000-2006	-2.0** (-3.1 - -0.8)	2006-2012	1.9*** (1.2 - 2.6)	2012-2019		
Iceland	1.0* (0.3 - 1.7)	1.6** (0.8 - 2.4)	2000-2007	-1.5 (-4.4 - 1.5)	2007-2011	1.7*** (1.1 - 2.4)	2011-2019		
Ireland	-0.2 (-0.5 - 0.2)	-0.8*** (-1.2 - -0.4)	2000-2012	0.9* (0.1 - 1.8)	2012-2019				
Israel	0.2 (-0.2 - 0.7)	1.2** (0.5 - 1.9)	2000-2006	-1.3 (-3.3 - 0.7)	2006-2010	0.3 (-0.1 - 0.6)	2010-2019		
Italy	-0.4* (-0.7 - -0.2)	-0.4** (-0.6 - -0.2)	2000-2007	0.4 (-1.1 - 1.9)	2007-2010	-1.2*** (-1.6 - -0.9)	2010-2016	0.4 (-0.3 - 1.2)	2016-2019
Japan	-0.5* (-0.7 - -0.3)	-1.1*** (-1.3 - -0.9)	2000-2009	-0.3 (-0.6 - 0.1)	2009-2016	0.7 (-0.3 - 1.8)	2016-2019		
Korea	0.7*** (0.5 - 0.8)	0.7*** (0.5 - 0.8)	2000-2019						
Latvia	1.1* (0.1 - 2.0)	2.7*** (2.2 - 3.2)	2000-2009	-1.5 (-4.1 - 1.1)	2009-2013	3.8 (-1.5 - 9.3)	2013-2016	-2.7* (-5.2 - -0.2)	2016-2019
Lithuania	0.7 (-0.3 - 1.7)	2.9*** (2.2 - 3.5)	2000-2008	-4.3 (-9.6 - 1.3)	2008-2011	2.1** (0.9 - 3.5)	2011-2017	-4.3 (-9.6 - 1.3)	2017-2019

Luxembourg	0.4<br>(-0.1 - 0.8)	3.8**<br>(1.3 - 6.4)	2000-<br>2002	0.0<br>(-0.4 - 0.5)	2002-<br>2009	1.9<br>(-0.6 - 4.5)	2009-<br>2012	-0.9***<br>(-1.2 - -0.6)	2012-<br>2019
Mexico	0.3*<br>(0.2 - 0.4)	0.1<br>(0.0 - 0.2)	2000-<br>2011	0.5***<br>(0.3 - 0.7)	2011-<br>2019				
Netherlands	0.1<br>(-0.1 - 0.4)	0.1<br>(-0.1 - 0.4)	2000-<br>2019						
New<br>Zealand	0.3*<br>(0.1 - 0.5)	0.3*<br>(0.1 - 0.5)	2000-<br>2019						
Norway	0.5<br>(-0.1 - 1.2)	-0.1<br>(-0.8 - 0.6)	2000-<br>2006	2.4**<br>(1.1 - 3.7)	2006-<br>2011	-1.8<br>(-5.5 - 2.1)	2011-<br>2014	0.8<br>(-0.1 - 1.7)	2014-<br>2019
Poland	0.2<br>(0.0 - 0.5)	-0.2<br>(-1.0 - 0.5)	2000-<br>2006	0.5***<br>(0.2 - 0.7)	2006-<br>2019				
Portugal	0.2<br>(-0.3 - 0.8)	0.3<br>(0.0 - 0.6)	2000-<br>2010	-1.5<br>(-5.1 - 2.1)	2010-<br>2013	1.1**<br>(0.5 - 1.7)	2013-<br>2019		
Slovak<br>Republic	-0.3**<br>(-0.5 - -0.1)	-0.3**<br>(-0.5 - -0.1)	2000-<br>2019						
Slovenia	-0.2**<br>(-0.3 - -0.1)	-0.2**<br>(-0.3 - -0.1)	2000-<br>2019						
Spain	-0.2*<br>(-0.5 - 0.0)	-0.7***<br>(-0.9 - -0.5)	2000-<br>2013	0.8*<br>(0.1 - 1.4)	2013-<br>2019				
Sweden	0.3*<br>(0.0 - 0.5)	3.2*<br>(0.6 - 6.0)	2000-<br>2002	-0.1<br>(-0.2 - 0.0)	2002-<br>2019				
Switzerland	0.1<br>(-0.1 - 0.3)	-0.7<br>('-1.5 - 0.1)	2000-<br>2005	0.4***<br>(0.2 - 0.6)	2005-<br>2019				
Türkiye	0.3<br>(0.0 - 0.7)	-0.3**.<br>(-0.6 - -0.1)	2000-<br>2009	1.9<br>(-0.6 - 4.4)	2009-<br>2012	0.5**<br>(0.2 - 0.9)	2012-<br>2019		
United<br>Kingdom	0.4*<br>(0.2 - 0.6)	1.5**<br>(0.5 - 2.6)	2000-<br>2003	0.0<br>(-0.2 - 0.1)	2003-<br>2014	0.6*<br>(0.1 - 1.1)	2014-<br>2019		
United<br>States	0.1<br>(-0.1 - 0.2)	0.1<br>(-0.1 - 0.4)	2000-<br>2007	-1.5**<br>(-2.3 - -0.6)	2007-<br>2011	0.8***<br>(0.6 - 0.9)	2011-<br>2019		

*: p<.05; **: p<0.01; ***: p<.001

Supplementary Table S2. Results of joinpoint regression models regarding protein supply of 38 OECD member states between 2000-2019.
