# Supplementary material for: Assessing the per Capita Food Supply Trends of 38 OECD Countries between 2000 and 2019—A Joinpoint Regression Analysis
Source: Life (Basel). 2023 Apr 27;13(5):1091. doi: 10.3390/life13051091 (PMC10222698; doi:10.3390/life13051091)
Supplement: Supplementary file 1 [file life-13-01091-s001.zip › Table S3.pdf]

| Country | APC (95%CI) | Trends | | | | | | | |
|---|---|---|---|---|---|---|---|---|---|
| | | Trend 1 | | Trend 2 | | Trend 3 | | Trend 4 | |
| | | AAPC (95%CI) | Period | AAPC (95%CI) | Period | AAPC (95%CI) | Period | AAPC (95%CI) | Period |
| Australia | 0.7* (0.3 - 1.0) | 0.7*** (0.5 - 1.0) | 2000-2008 | 2.4* (0.0 - 4.8) | 2008-2011 | -0.1 (-0.3 - 0.2) | 2011-2019 | | |
| Austria | -0.1 (-0.4 - 0.2) | -0.9* (-1.6 - -0.3) | 2000-2004 | 1.4 (-0.8 - 3.5) | 2004-2007 | -0.2* (-0.3 - 0.0) | 2007-2019 | | |
| Belgium | 0.1* (0.0 - 0.2) | -0.1 (-0.2 - 0.0) | 2000-2009 | 0.3*** (0.1 - 0.4) | 2009-2019 | | | | |
| Canada | 0.1 (0.0 - 0.2) | -0.1*** (-0.2 - -0.1) | 2000-2015 | 0.8* (0.3 - 1.3) | 2015-2019 | | | | |
| Chile | 0.3*** (0.3 - 0.4) | 0.3*** (0.3 - 0.4) | 2000-2019 | | | | | | |
| Colombia | 0.3 (-0.1 - 0.7) | -0.2 (-0.5 - 0.0) | 2000-2011 | 2.1* (0.3 - 3.8) | 2011-2015 | 0.0 (-1.0 - 1.2) | 2015-2019 | | |
| Costa Rica | 0.4 (0.0 - 0.8) | -0.8 (-2.0 - 0.4) | 2000-2003 | 1.4 (-1.0 - 3.9) | 2003-2006 | 0.1 (-0.7 - 0.4) | 2006-2012 | 0.9*** (0.6 - 1.3) | 2012-2019 |
| Czech Republic | 0.4* (0.2 - 0.5) | 2.6*** (1.8 - 3.4) | 2000-2003 | -0.3*** (-0.4 - -0.2) | 2003-2015 | 0.8* (0.3 - 1.3) | 2015-2019 | | |
| Denmark | 0.1 (-0.1 - 0.4) | 0.5* (0.3 - 0.8) | 2000-2006 | -1.0 (-2.6 - 0.7) | 2006-2009 | 0.2* (0.1 - 0.4) | 2009-2019 | | |
| Estonia | 0.2 (0.0 - 0.5) | 0.1 (-0.9 - 1.2) | 2000-2003 | 1.5*** (1.0 - 2.0) | 2003-2009 | -0.9*** (-1.2 - -0.5) | 2009-2016 | 0.4 (-0.7 - 1.4) | 2016-2019 |
| Finland | 0.3*** (0.3 - 0.4) | 0.3*** (0.3 - 0.4) | 2000-2019 | | | | | | |
| France | -0.1 (-0.3 - 0.0) | -0.3*** (-0.5 - -0.2) | 2000-2014 | 0.5 (-0.2 - 1.1) | 2014-2019 | | | | |
| Germany | 0.3* (0.2 - 0.4) | 0.7*** (0.4 - 0.9) | 2000-2007 | 0.1** (0.0 - 0.2) | 2007-2019 | | | | |
| Greece | -0.4 ('-0.8 - 0.0) | 0.5 (-0.3 - 1.4) | 2000-2004 | -1.8 (-4.3 - 0.9) | 2004-2007 | -0.3** (-0.5 - -0.2) | 2007-2019 | | |
| Hungary | 0.4 (-0.1 - 0.9) | -2.1 (-4.9 - 0.8) | 2000-2002 | 2.5 (-0.5 - 5.6) | 2002-2005 | -1.2*** (-1.7 - -0.7) | 2005-2012 | 1.8*** (1.4 - 2.2) | 2012-2019 |
| Iceland | 0.8* (0.5 - 1.1) | 1.4*** (1.1 - 1.6) | 2000-2007 | -0.4 (-1.0 - 0.2) | 2007-2012 | 1.6*** (1.0 - 2.2) | 2012-2017 | -0.1 (-1.9 - 1.7) | 2017-2019 |
| Ireland | 0.2 (-0.1 - 0.4) | -1.2** (-1.9 - -0.6) | 2000-2005 | 0.4* (0.1 - 0.8) | 2005-2013 | 1.1*** (0.6 - 1.6) | 2013-2019 | | |
| Israel | 0.0 (-0.3 - 0.3) | 1.6 (-1.0 - 4.4) | 2000-2002 | -0.6* (-1.2 - 0.0) | 2002-2008 | 0.1 (-0.1 - 0.2) | 2008-2019 | | |

| | | | | | | | | | |
|---|---|---|---|---|---|---|---|---|---|
| Italy | -0.3*** (-0.4 - -0.2) | -0.3*** (-0.4 - -0.2) | 2000-2019 | | | | | | |
| Japan | -0.4* (-0.7 - -0.1) | -0.6*** (-0.7 - -0.4) | 2000-2007 | -1.5* (-3.0 - 0.0) | 2007-2010 | 0.9 (-0.6 - 2.4) | 2010-2013 | -0.3* (-0.6 - -0.1) | 2013-2019 |
| Korea | 0.6* (0.4 - 0.8) | 0.1 (-0.2 - 0.4) | 2000-2006 | 1.6** (0.7 - 2.4) | 2006-2010 | 0.5*** (0.4 - 0.7) | 2010-2019 | | |
| Latvia | 0.8* (0.6 - 1.1) | 3.8*** (2.2 - 5.4) | 2000-2002 | 1.5** (0.7 - 2.3) | 2002-2006 | -0.3 (-0.6 - 0.1) | 2006-2012 | 0.5*** (0.3 - 0.7) | 2012-2019 |
| Lithuania | 0.4* (.1 - 0.8) | 1.7*** (1.4 - 2.0) | 2000-2007 | -2.1 (-4.3 - 0.1) | 2007-2010 | 0.3* (0.1 - 0.5) | 2010-2019 | | |
| Luxembourg | 0.0 (0.0 - 0.1) | 0.1*** (0.1 - .2) | 2000-2013 | -0.8** (-1.3 - -0.3) | 2013-2016 | 0.5** (0.2 - 0.7) | 2016-2019 | | |
| Mexico | 0.2*** (0.1 - 0.2) | 0.2*** (0.1 - 0.2) | 2000-2019 | | | | | | |
| Netherlands | 0.1 (0.0 - 0.2) | -0.8* (-1.4 - -0.2) | 2000-2003 | 0.1* (0.0 - 0.2) | 2003-2016 | 1.2** (0.6 - 1.8) | 2016-2019 | | |
| New Zealand | 0.1 (0.0 - 0.2) | 0.1 (0.0 - 0.2) | 2000-2019 | | | | | | |
| Norway | 0.1 (-0.2 - 0.4) | 1.1 (-0.9 - 3.2) | 2000-2002 | 0.1 (-0.1 - 0.3) | 2002-2012 | -0.7* (-1.3 - 0.0) | 2012-2017 | 1.0 (-1.1 - 3.1) | 2017-2019 |
| Poland | 0.2 (-0.2 - 0.5) | -0.3* (-0.5 - -0.1) | 2000-2008 | 1.2 (-0.8 - 3.1) | 2008-2011 | -0.6 (-1.5 - 0.4) | 2011-2015 | 1.1** (0.5 - 1.8) | 2015-2019 |
| Portugal | -0.1 (-0.3 - 0.0) | 0.0 (-0.2 - 0.1) | 2000-2008 | -0.7** (-1.0 - -0.4) | 2008-2014 | 0.4* (0.0 - 0.7) | 2014-2019 | | |
| Slovak Republic | 0.3* (0.0 - 0.5) | 0.3* (0.1 - 0.6) | 2000-2009 | -0.5 (-1.1 - 0.1) | 2009-2015 | 1.3** (0.5 - 2.2) | 2016-2019 | | |
| Slovenia | 0.2* (0.1 - 0.3) | 0.4** (0.2 - 0.6) | 2000-2009 | 0.0 (-0.2 - 0.2) | 2009-2019 | | | | |
| Spain | 0.0 (-0.2 - 0.2) | -0.9*** (-1.3 - -0.5) | 2000-2005 | -0.2* (-0.4 - 0.0) | 2005-2014 | 1.3*** (0.9 - 1.7) | 2014-2019 | | |
| Sweden | 0.1 (0.0 - 0.3) | 0.3 (0.0 - 0.5) | 2000-2004 | -0.5 (-1.4 - 0.4) | 2004-2007 | 0.5*** (0.3 - 0.7) | 2007-2013 | -0.1 (-0.2 - 0.1) | 2013-2019 |
| Switzerland | -0.1 (-0.2 - 0.1) | 0.1 (-0.1 - 0.3) | 2000-2010 | -0.3* (-0.5 - 0.0) | 2010-2019 | | | | |
| Türkiye | 0.1 (0.0- 0.3) | -0.6 (-1.4 - 0.2) | 2000-2003 | 0.3*** (0.2 - 0.3) | 2003-2019 | | | | |
| United Kingdom | 0.0 (-0.1 - 0.1) | 0.5* (0.0 - 1.0) | 2000-2004 | -0.1** (-0.2 - -0.1) | 2004-2019 | | | | |
| United States | 0.2 (0.0 - .3) | 0.5* (0.1 - 0.9) | 2000-2005 | -1.1** (-1.6 - -0.5) | 2005-2010 | 0.7*** (0.5 - 0.8) | 2010-2019 | | |

*: p<.05; **: p<0.01; ***: p<.001

**Supplementary Table S3.** Results of joinpoint regression models regarding calories supply of 38 OECD member states between 2000-2019.
